# 2043: Pharmacokinetic prediction of paclitaxel-induced peripheral neuropathy

**DOI:** 10.1017/cts.2017.114

**Published:** 2018-05-10

**Authors:** Daniel L. Hertz, Kelley M. Kidwell, Kiran Vangipuram, Duxin Sun, N. Lynn Henry

## Abstract

OBJECTIVES/SPECIFIC AIMS: Peripheral neuropathy is the dose limiting toxicity of paclitaxel treatment. Paclitaxel pharmacokinetics (PK), specifically the Cmax and amount of time the concentration remains above 0.05 µM (Tc>0.05), have been associated with occurrence of severe, clinician-documented neuropathy. The objective of this study was to confirm that paclitaxel PK predicts progression of patient-reported neuropathy. METHODS/STUDY POPULATION: This observational trial enrolled breast cancer patients receiving weekly 1-hour paclitaxel infusions (80 mg/m^2^×12 cycles) at the University of Michigan Comprehensive Cancer Center. Paclitaxel concentration was measured via LC/MS in plasma samples collected at the end of (Cmax) and 16–24 hours after (Tc>0.05) first infusion. Patient-reported neuropathy was collected (EORTC CIPN20) at baseline and each cycle. The rate of neuropathy severity increase per treatment cycle is being modeled for each patient. Cmax and Tc>0.05 values will be introduced into the model to confirm that PK independently contributes to neuropathy progression. RESULTS/ANTICIPATED RESULTS: PK and neuropathy data have been collected from 60 patients for ongoing analysis. Our initial model will characterize the expected severity of neuropathy after each cycle of paclitaxel treatment. The PK-neuropathy model will include either PK parameter to validate their contribution to the progression of neuropathy severity during treatment. We anticipate, based on our preliminary analysis of the first 16 patients, that both PK parameters will significantly contribute to the model but Tc>0.05 will be more strongly associated with neuropathy progression. DISCUSSION/SIGNIFICANCE OF IMPACT: This project will generate a model that can be used to predict a patient’s neuropathy severity throughout treatment using a single, conveniently collected and easily measured PK sample during their first cycle. The next steps of this project include identifying genetic and metabolomic biomarkers that predict which patients experienced more severe neuropathy than would be anticipated based on their paclitaxel PK, and a planned interventional trial of personalized paclitaxel dosing to enhance efficacy and/or prevent neuropathy.

